# Molecular Alliance of *Lymantria dispar* Multiple Nucleopolyhedrovirus and a Short Unmodified Antisense Oligonucleotide of Its Anti-Apoptotic IAP-3 Gene: A Novel Approach for Gypsy Moth Control

**DOI:** 10.3390/ijms18112446

**Published:** 2017-11-17

**Authors:** Volodymyr V. Oberemok, Kateryna V. Laikova, Aleksei S. Zaitsev, Maksym N. Shumskykh, Igor N. Kasich, Nikita V. Gal’chinsky, Viktoriya V. Bekirova, Valentin V. Makarov, Alexey A. Agranovsky, Vladimir A. Gushchin, Ilya V. Zubarev, Anatoly V. Kubyshkin, Iryna I. Fomochkina, Mikhail V. Gorlov, Oleksii A. Skorokhod

**Affiliations:** 1 Taurida Academy, Department of Biochemistry, V.I. Vernadsky Crimean Federal University, Simferopol 295007, Republic of Crimea; genepcr@mail.ru (V.V.O.); zaycevfl@mail.ru (A.S.Z.); hinon@list.ru (M.N.S.); alchamy1994@mail.ru (N.V.G.); viktoriya.bekirova@ya.ru (V.V.B.); 2 Medical Academy, Department of Biochemistry, V.I. Vernadsky Crimean Federal University, Simferopol 295006, Republic of Crimea; botan_icus@mail.ru; 3 Medical Academy, Department of Pathological Anatomy, V.I. Vernadsky Crimean Federal University, Simferopol 295006, Republic of Crimea; igrkas@gmail.com; 4 Belozersky Institute of Physico-Chemical Biology, Lomonosov Moscow State University, Moscow 119991, Russia; makarovvalentine@gmail.com; 5 Department of Virology, Lomonosov Moscow State University, Moscow 119991, Russia; aaa@genebee.msu.su (A.A.A.); vladimir.a.gushchin@gamaleya.org (V.A.G.); 6 Center of Bioengineering, Russian Academy of Sciences, Moscow 117312, Russia; 7 Translational Biomedicine Laboratory, N. F. Gamaleya Federal Research Centre for Epidemiology and Microbiology, Moscow 123098, Russia; 8 Institute of Natural Sciences, Ural Federal University, Chelyabinsk 620083, Russia; ilyamitozubarev@gmail.com; 9 Medical Academy, Department of General and Clinical Pathophysiology, V.I. Vernadsky Crimean Federal University, Simferopol 295006, Republic of Crimea; kubyshkin_av@mail.ru (A.V.K.); fomochkina_i@mail.ru (I.I.F.); 10 Department of Polymer Chemistry, Mendeleev University of Chemical Technology of Russia, Moscow 125047, Russia; mikgorlov@gmail.com; 11 University of Torino, 10124 Torino, Italy; 12 Department of Molecular Biosciences, The Wenner-Gren Institute, Stockholm University, 10691 Stockholm, Sweden

**Keywords:** baculovirus infection, gypsy moth *Lymantria dispar*, multiple nucleopolyhedrovirus IAP (inhibitor-of-apoptosis) genes, RING (really interesting new gene), DNA insecticides, insect pest management

## Abstract

Baculovirus IAP (inhibitor-of-apoptosis) genes originated by capture of host genes. Unmodified short antisense DNA oligonucleotides (oligoDNAs) from baculovirus IAP genes can down-regulate specific gene expression profiles in both baculovirus-free and baculovirus-infected insects. In this study, gypsy moth (*Lymantria dispar*) larvae infected with multiple nucleopolyhedrovirus (LdMNPV), and LdMNPV-free larvae, were treated with oligoDNA antisense to the RING (really interesting new gene) domain of the LdMNPV *IAP-3* gene. The results with respect to insect mortality, biomass accumulation, histological studies, RT-PCR, and analysis of DNA apoptotic fragmentation suggest that oligoRING induced increased apoptotic processes in both LdMNPV-free and LdMNPV-infected insect cells, but were more pronounced in the latter. These data open up possibilities for promising new routes of insect pest control using antisense phosphodiester DNA oligonucleotides.

## 1. Introduction

The use of unmodified nucleic acids as insecticides looks very promising, since they can work selectively, are subject to fast biodegradation in ecosystems (in contrast to the majority of conventional chemical insecticides), and the commercial synthesis of nucleic acids in vitro is becoming more affordable.

RNA interference (RNAi) and the use of double-stranded RNA (dsRNA) fragments is a viable possibility for use in the control of insect pests, particularly lepidopterans [1,2]. However, this approach has several apparent drawbacks. The efficacy of RNAi action in lepidopteran insects could be compromised because: (i) some insect genes are resistant to RNAi [3], (ii) the high cost of RNA substrate synthesis, and (iii) the problem of target delivery under field conditions [2]. Moreover, the dsRNAs in cells are cleaved into numerous, very short (21–23 nucleotide) siRNAs with abundant direct sequence matches among the genomes of non-target organisms, seriously lowering the selectivity of this approach [4].

In 2008, we proposed the topical application of nucleic acids for control of phytophagous insects. Application of short pest-specific unmodified antisense DNA fragments (DNA insecticides) against the gypsy moth *Lymantria dispar*, a major insect pest of hardwood trees, was followed by significant larval mortality [5]. Three years later, Wang et al. [6] topically applied dsRNA fragments specific for the Asian corn borer *Ostrinia furnacalis*, which was also followed by significant larval mortality [6]. Previously, it had been thought that oral administration was the only possible way to deliver double-stranded RNAs to target tissues, other than injection, as the insect midgut is not protected by chitin integument [2]. The induction of insect mortality by topical application of single-stranded DNA or double-stranded RNA could be explained by their passage into interior tissues via the tracheal system, which is not covered by chitinous exoskeleton [1] or via diffusion through the soft thin cuticle of young instars.

We see a number of advantages that make DNA insecticides a more promising approach against lepidopterans at the larval stage than elaborate RNA preparations. First, short (~18–20 nucleotides long) insect-specific DNA insecticides are more affordable compared to relatively long double-stranded RNA fragments. Second, gene silencing achieved by feeding or injection of double-stranded RNA requires high concentrations for success. This issue could be resolved with topical application of DNA insecticides based on short antisense DNA fragments acting in substantially lower concentrations. In most studies, the “standard” amount of double-stranded RNA injected to achieve high levels of RNAi (but not death) in insects varies between 1 and 100 µg per mg of biomass [3]. For comparison, in experiments with DNA insecticides, we topically applied 3–30 pmol of viral 18-nucleotide-long DNA fragments per gypsy moth larva, corresponding to approximately 1.8–180 ng of DNA per mg of larval biomass. Therefore, single-stranded DNA insecticides work in substantially lower concentrations; accordingly, this approach may be cheaper for insect pest control compared with RNA preparations. It may be impossible to use DNA insecticides against secretive insects and adult beetles, because elytra could provide some protection from a contact insecticide. Nevertheless, DNA insecticides look very suitable for use in insect pest control of non-secretive lepidopteran pests at the larval stage, especially during early larval instars, when the insects’ exoskeletons are thin. Third, very short 18 nucleotides long pest-specific antisense DNA fragments (DNA insecticide) will not be cleaved in the cells of non-target organisms, unlike relatively long dsRNAs, which are diced into very short siRNAs that may silence non-target genes [4]. Lastly, the presence of the 2′-OH group makes the hydrolysis of RNA much more facile than hydrolysis of DNA [7]. Thus, DNA insecticides will be more stable than RNA preparations under natural conditions, and thus, can cause a greater insecticidal effect before they are degraded.

According to results from our most recent research, unmodified antisense DNA oligonucleotides (oligoDNAs) of the LdMNPV (*Lymantria dispar* multiple nucleopolyhedrovirus) *IAP-3* (inhibitor-of-apoptosis) gene have pronounced insecticidal effects on LdMNPV-free gypsy moths [5,8,9]. Baculoviruses are insect pathogenic viruses used as biological control agents. Baculoviruses encode inhibitors-of-apoptosis (IAP) proteins, which are classified into 5 groups, IAP-1–5, based on their sequence homology. Most of the baculovirus IAPs with anti-apoptotic functions belong to the *IAP-3* group, with certain exceptions [10]. All IAP genes isolated from different baculoviruses display two distinct structural features. The first is the presence of amino-terminal repeats of an amino acid sequence termed the baculovirus IAP repeat (BIR). The second is a zinc binding domain known as a RING (really interesting new gene) finger [11]. BIR contains approximately 70 amino acids that coordinate with a zinc ion via histidine and cysteine residues [12]. It has been shown that the BIR domain is necessary for the interaction of IAP proteins with diverse pro-apoptotic factors, including invertebrate death inducers, and vertebrate and invertebrate members of the caspase family of proteases [10]. RING domains are characterized by the presence of 6 or 7 cysteines and 1 or 2 histidines that form a cross brace architecture and coordinate two zinc ions. RING domains often function as modules that confer ubiquitin protein ligase (E3) activity and, in conjunction with an ubiquitin activity enzyme (E1) and an ubiquitin conjugating enzyme (E2), catalyze the transfer of ubiquitin to target proteins. All known RING-containing IAPs have E3 activity; the range of substrates includes molecules involved in apoptosis and signaling, as well as the RING-containing IAP themselves in a homo- or heterotypic fashion [12]. A typical animal IAP protein includes three homologous BIR domains and a RING domain near the C-terminus. Both sequence and phylogenetic analyses have revealed that, in the case in which the viral IAP has only one BIR domain, it is homologous to the third BIR domain of animal IAP. For example, in LdMNPV the *IAP-3* gene encodes a 155-amino acid long protein, where the region from the 6th to 71st amino acids belongs to the BIR domain, and the region from the 105th to 149th amino acids belongs to the RING domain [13]. Thus, the presence of homologous IAP in *Lymantria dispar*, as was supposed in our previous studies, is a good target for apoptosis induction after oligoDNAs application. Moreover, the baculovirus IAPs bear a striking resemblance to the cellular IAPs carried by the host insects that they infect [14]. Cellular IAPs are a highly conserved family of survival factors that regulate developmental- and stress-induced apoptosis, as well as inflammation, the cell cycle, and some signaling processes [12,15].

Although the exact mechanism of action of DNA insecticides is currently under study, we have a lot of evidence that DNA insecticides work in a manner similar to unmodified [16] and modified antisense oligonucleotides [17] used in medicine, generating antisense effects through RNase H-dependent mechanism [18,19]. The mechanism of action of antisense oligoDNAs from IAP genes is thought to be the same as that of antisenseRNase H-dependent oligonucleotides. RNase H is a ubiquitous enzyme that hydrolyzes the RNA strand of an RNA/DNA duplex. Oligonucleotide-assisted RNase H-dependent reduction of targeted RNA expression can be quite efficient, reaching 80–95% down-regulation of protein and mRNA expression [10,16]. In the case of IAP targeting, down-regulation of the anti-apoptosis protein expression leads to increased apoptotic processes in cells and subsequent death of the insect. Historically, we have chosen RING as a target because the RING domain is a highly conserved segment of insect and baculovirus IAP genes. Using the GenBank NIH genetic sequence database, we searched for the conserved antisense segment inside the RING domain fragment in the LdMNPV *IAP-3* gene and decided to use the 5′-CGACGTGGTGGCACGGCG-3′ sequence. This fragment is found in all LdMNPV genomes represented in GenBank and is considered to be well conserved. We decided to use this conserved fragment as oligoDNA (oligoRING) for the treatments, and the presence of 4 CG motifs in oligoRING suggests us to use oligoCpG, a non-specific TLR-9 inducer [20], as a control. In the same vein, DNA insecticides could resolve or improve upon an important insecticide resistance problem: use of short single-stranded fragments of highly conserved segments of insect host anti-apoptosis genes will result in slower development of resistance to these insecticides, because it is known that the potential mutations that change the target anti-apoptosis genes occur at a very low rate in the conserved parts of the genes.

It is important to note that the use of phosphodiester oligonucleotides in mammalian tissues is limited, as they are rapidly degraded by intracellular endonucleases and exonucleases [16]. However, the activity of insect enzymes (e.g., esterases) is weaker than that of mammals [21]. This paves the way for the prospective use of unmodified antisense oligonucleotides as a selective tool for insect pest control [10,19,22].

Our particular attention will be devoted to LdMNPV-infected larvae, a group widely distributed among gypsy moth populations [23,24]. Baculovirus infections in lepidopterans may cause difficulties in silencing with nucleic acids, particularly dsRNA [3,25]. In this study we investigate further the insecticidal effect of the oligoRING fragment antisense to RING (really interesting new gene) domain of the LdMNPV *IAP-3* gene on both non-infected and LdMNPV-infected gypsy moth larvae, and show how to turn this possible weakness into a strength by applying this approach to insect pest management.

## 2. Results

### 2.1. Nucleopolyhedrovirus (LdMNPV)-Free Gypsy Moth Larvae Grown From Eggs in the Laboratory May Not Be Sufficiently Sensitive to the Antisense oligoRING Fragment

#### 2.1.1. LdMNPV Contamination Exclusion

It is known that LdMNPV is widespread among gypsy moth populations and may be transmitted transovarially [23,24]. Naturally occurring LdMNPV epizootics often run their course and eventually control outbreaks among populations of gypsy moths in non-recreational forested areas [26]. Consequently, to exclude the presence of the virus in experimental insects, the 1st instar gypsy moth larvae (hatched from eggs in the lab) were checked for the LdMNPV infection using PCR with two oligonucleotide primers specific to the viral capsid gene p39 [9]. All tested larvae were free from the baculovirus (see Supplemental Figure S1).

#### 2.1.2. The Effect of the DNA oligonucleotides (oligoDNAs) on the LdMNPV-Free Gypsy Moth Larvae

The 1st instar gypsy moth larvae were treated with oligoDNAs, namely oligoRING, a DNA oligonucleotide complementary to the mRNA sequence of the LdMNPV anti-apoptosis *IAP-3* gene, and oligoCpG, a non-specific TLR-9 inducer [27]. During the 14 days of observation, treatment with the oligoRING did not show any statistically significant effect (*p* > 0.05) on the viability of baculovirus-free larvae compared with those of water- and oligoCpG-treated controls, and mortality in all groups of the experiment comprised 5–8%.

#### 2.1.3. Search for the oligoRING (Really Interesting New Gene Oligonucleotides) Target mRNA

Even though the antisense oligoRING fragment did not have an insecticidal effect on the larvae we tested, it is important to know whether gypsy moth cells have the target mRNA complementary to that of the oligoRING. We tested mRNAs of LdMNPV-free gypsy moth larvae to find out whether gypsy moth cells contain a host anti-apoptosis gene homologous to the LdMNPV *IAP-3* gene. Of note, recently we cloned part of the total genomic DNA of the gypsy moth using fragments of the BIR and RING domains of LdMNPV *IAP-3* gene as primers. We found that it had an overlap with the corresponding part of the database reported for the LdMNPV *IAP-3* gene and *Lymantria dispar IAP-1* mRNA for an inhibitor of apoptosis protein with high cover by query [8]. This allowed us to assume that the larvae had expressed the gene with the mRNA complementary to the oligoRING, and thus, we cloned a part of a new gypsy moth anti-apoptosis gene.

**Figure 1:**
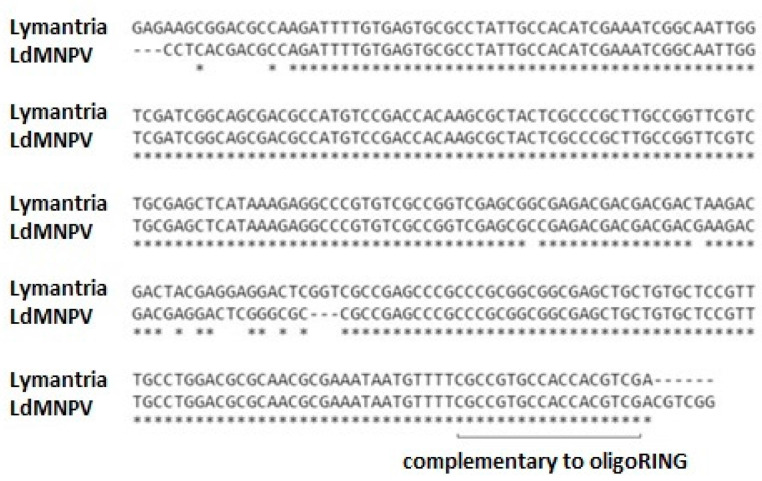
Alignment of the sequenced DNA fragments of gypsy moth and nucleopolyhedrovirus (LdMNPV) *IAP-3* gene fragments performed using ClustalW 2.0.3; identities found by simple comparison of the sequences are marked with *.

To evaluate the possibility that the action of the oligoRING occurred through the mechanisms characteristic for antisense RNase H-dependent oligonucleotides [10], we used PCR with oligoBIR and oligoRING sequences of the LdMNPV *IAP-3* gene as primers and revealed one specific fragment of cDNA derived from the total mRNA of the gypsy moth (Supplemental Figure S2). This fragment from each insect DNA spectrum was chosen for DNA purification and further DNA sequencing. The sequenced cDNA fragment had a high cover by query (>90%) with the sequenced LdMNPV *IAP-3* gene fragment (Figure 1), confirming that in experimental larvae we found mRNA of a homologous anti-apoptosis gene of the gypsy moth.

Independently, by analyzing mRNA of non-infected *Lymantria dispar* larvae reversed in cDNA and using the TBLASTX database, we demonstrated that the *Lymantria dispar IAP-1* gene (described by the Sugiyama group in 2012 and available at http://www.ncbi.nlm.nih.gov/nuccore/409924403) has 55% cover by query with the sequenced cDNA fragment. For this reason, we believe that we have found, not the *IAP-1* gene, but a new *Lymantria dispar* anti-apoptotic gene, which we call the *IAP-Z* gene. The LdMNPV *IAP-3* gene is highly similar to the *IAP-Z* gene (Figure 1). Logically, if we were to find the host IAP gene that closely resembles the LdMNPV IAP gene, it also could play a crucial role in the apoptosis-antiapoptosis system, as both organisms (the virus and the host) use the similar IAPs for the same purpose. For this reason, the baculovirus IAPs homologous to the host IAPs are among the most promising prospects in the development of DNA insecticides against gypsy moth, and thus, we tried to target them.

**Figure 2:**
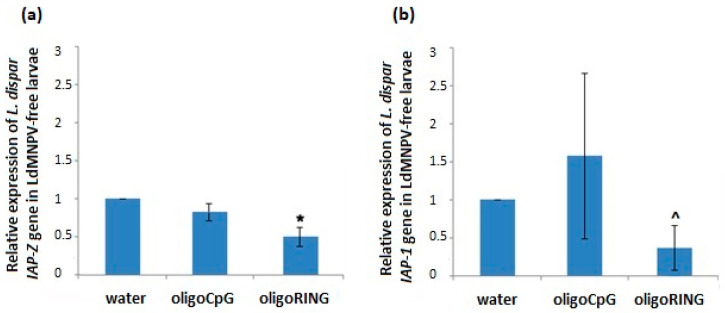
Expression of gypsy moth *IAP-Z* and *IAP-1* genes after treatment with oligoDNAs. (**a**) Treatment of LdMNPV-free larvae with oligoRING (RING column) leads to significant down-regulation of host *IAP-Z* gene expression on the 14th day. Values represent means and standard errors of mRNA expression for 3 replicates relative to the water-treated control group. The significant difference between the oligoRING group and the water-treated control group is indicated by * when *p* < 0.05; (**b**) Treatment of LdMNPV-free gypsy moth larvae with oligoRING (RING column) leads to markedly decreased expression of the host *IAP-1* gene on the 14th day. Values represent means and standard errors of mRNA expression for 3 replicates relative to the control water-treated control group. The significant difference between the oligoRING group and the water-treated control group is indicated by ^ when *p* < 0.12.

Using oligo*IAP-Z* (see Methods for the sequence) and oligoRING primers, we checked whether the target host anti-apoptosis gene sequenced by us is down-regulated in gypsy moth cells after application of the antisense oligoRING fragment. We evaluated gene expression of both the target host *IAP-Z* gene and the non-target host *IAP-1* gene on the 14th day post-treatment. The results showed that in LdMNPV-free larvae, the antisense oligoRING fragment caused a significant down-regulation (2.04 ± 0.29-fold stronger) of the host target *IAP-Z* gene compared with that of control (*p* < 0.05) (Figure 2a). Further, they showed a substantial but not significant decrease of host *IAP-1* gene expression, which we believe to be connected with the down-regulation of the *IAP-Z* gene (Figure 2b). Control fragment oligoCpG showed an insignificant difference compared with control for both host IAP genes. Thus, the oligoRING fragment acts as an antisense RNase H-dependent oligonucleotide [16].

**Figure 3:**
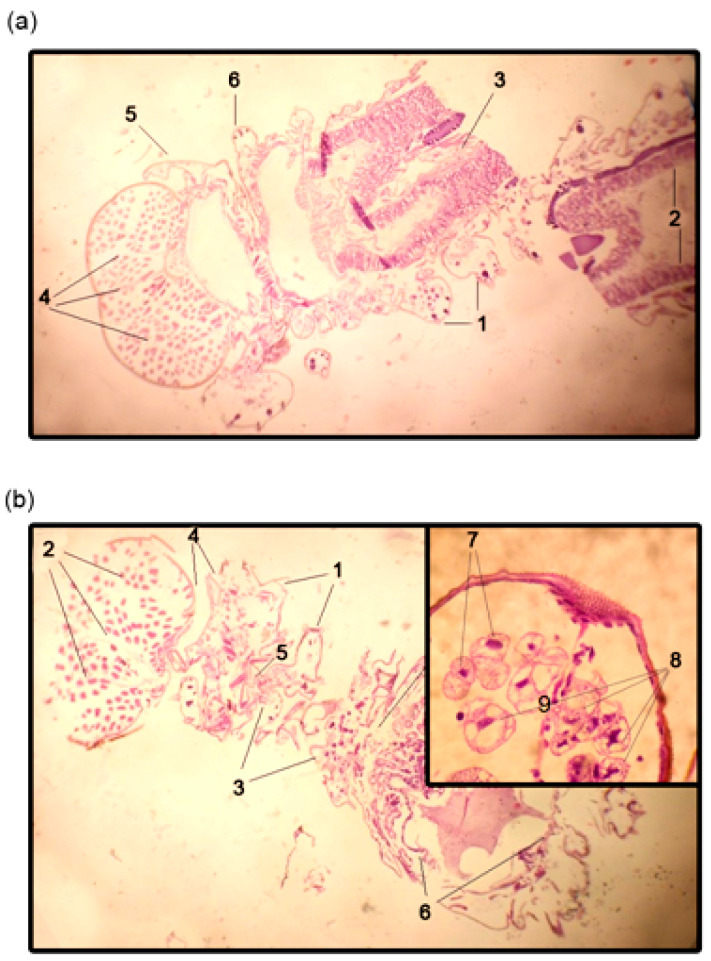
Light microscopy of the gypsy moth larvae histological slides. (**a**) Control sample (water-treated control): 1—outer layers; 2—intestinal epithelium; 3—esophagus; 4—pigment cells and their septum; 5—mandible; 6—maxilla; (**b**) OligoRING-treated larvae showing pronounced apoptotic destruction of tissues: 1—outer layers; 2—pigment cells with destroyed septum; 3—separate clusters of epithelial cells of the digestive tube; 4—destroyed mouth apparatus; 5—heart; 6—anal section of the digestive tube; 7—normal cell; 8—apoptotic signatures (pyknosis, karyorrhexis, and involution of cells); 9—vacuolated cell. Magnification: ×40 for (**a**), (**b**) and ×80 for the zoomed fragment of (**b**).

On the 14th day, gypsy moth larvae treated with oligoDNAs were also sliced and studied under a light microscope. The most pronounced apoptotic patterns were observed in the outer covering tissue of oligoRING-treated larvae, including structural deformation, involution of cells, and condensation (pyknosis) and fragmentation (karyorrhexis) of nuclear material [28] (Figure 3).

Thus, the antisense oligoRING fragment triggered stronger apoptotic processes in LdMNPV-free gypsy moth larvae compared with observations in the oligoCpG and water-treated control groups.

### 2.2. Topical Application of oligoRING from the LdMNPV IAP-3 Gene Increases the Mortality Rate of LdMNPV-Infected Gypsy Moth Larvae “That Which Is Falling Should also Be Pushed”

#### 2.2.1. Evidence of Successful Infection with LdMNPV

Prior to experiments with LdMNPV-infected gypsy moth larvae, the larvae were checked for LdMNPV infection, as described in Methods. The level of LdMNPV infection among the tested larvae (20 individuals) was 50% on the 5th day post-infection (Supplemental Figure S3).

#### 2.2.2. Topical Treatment with the Antisense oligoRING Fragment Leads to Decreased Rate of Biomass Accumulation and an Increased Mortality Rate in LdMNPV-Infected Gypsy Moth Larvae

**Figure 4:**
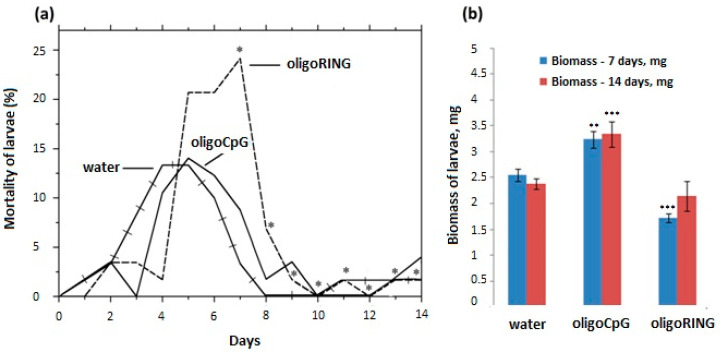
The oligoRING’s effect on the mortality and biomass accumulation of LdMNPV-infected gypsy moth larvae. (**a**) Non-cumulative curve of mortality of LdMNPV-infected larvae after treatment with oligoDNAs. The significant difference is indicated by * for *p* < 0.05. Each group (water-treated control, oligoCpG, and oligoRING) used 20 to 25 larvae per replicate. Each experiment was conducted in triplicate; (**b**) The mean body mass of LdMNPV-infected larvae after treatment with water, oligoCpG, or oligoRING is shown in mg. SE (standard errors) are given for three replicates. Significant difference: ** for *p* < 0.01; *** for *p* < 0.001.

LdMNPV-infected 1st instar gypsy moth larvae were treated with water, oligoRING, or oligoCpG. A significant increase in mortality of LdMNPV-infected larvae in the oligoRING group compared with the water-treated control group was observed on the 7th day after treatment (χ^2^ = 5.51; *p* < 0.05). On average, 53.3%, 50.9%, and 74.1% of the LdMNPV-infected larvae died in the groups treated with distilled water, oligoCpG, and oligoRING, respectively (Figure 4a). At the end of the experiment, the 14th day after treatment, treatment with the oligoRING increased the statistically significant effect on the mortality of LdMNPV-infected insects compared to that of the water-treated controls (χ^2^ = 9.63, *p* < 0.01) and oligoCpG controls (*p* < 0.05); the percentage of dead insects reached 63.3% (water), 61.4% (oligoCpG), and 87.9% (oligoRING), respectively (Table 1).

We also detected a significant reduction in biomass accumulation in the surviving larvae in the oligoRING group (1.72 ± 0.049 mg) compared with that in the water-treated control group (2.55 ± 0.072 mg) on the 7th day (*p* < 0.01) (Figure 4b). Obviously, thus, the oligoRING fragment triggered higher levels of cell apoptosis, which decreased the biomass of the LdMNPV-infected larvae [9]. On the 14th day, the average biomass of the oligoRING-treated larvae (2.14 ± 0.16 mg) became insignificant compared with that of controls (2.38 ± 0.06 mg). The control DNA oligonucleotide (oligoCpG) showed the opposite trend and significantly increased the rate of biomass accumulation in larvae on both the 7th and 14th days (*p* < 0.01). This indicates that different sequences of oligonucleotides can cause opposite effects on insects.

### 2.3. The oligoRING Significantly Decreases Expression of the Host IAP-1 Gene and Triggers Total Down-Regulation of Baculovirus IAP-3 and Host IAP-Z Genes in LdMNPV-Infected Gypsy Moth Larvae

**Figure 5:**
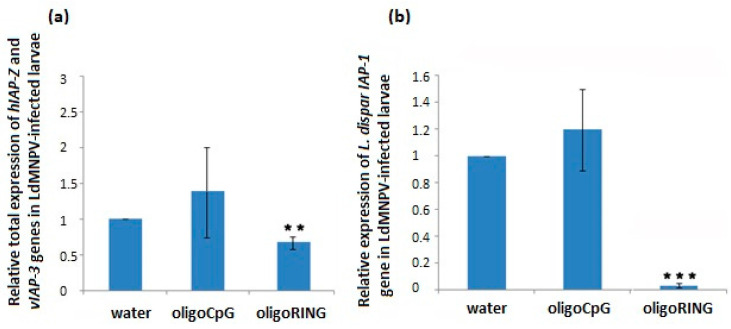
Expression of gypsy moth *IAP-1* gene and joint expression of baculovirus *IAP-3* and host *IAP-Z* genes after treatment with oligoDNAs. (**a**) Treatment of LdMNPV-infected larvae with oligoRING leads to significant down-regulation of total expression of *hIAP-Z* and *vIAP-3* genes on the 6th day. Values represent means and standard errors of mRNA expression for 3 replicates relative to the water-treated control group. The significant difference between oligoRING group vs. water control is indicated by ** when *p* < 0.01; (**b**) Treatment of LdMNPV-infected gypsy moth larvae with the oligoRING leads to a significant decrease in expression of the host *IAP-1* gene on the 14th day. Values represent means and standard errors of mRNA expression for 3 replicates relative to the water-treated control group. The significant difference between oligoRING group vs. the water-treated control group is indicated by *** when *p* < 0.001.

On the 6th day (LdMNPV-infected larvae mortality peak) after treatment with the oligoDNAs, we measured the joint expression of baculovirus *IAP-3* and host *IAP-Z* genes (Figure 5a), and on 14th day, the expression of the host *IAP-1* gene (Figure 5b). In LdMNPV-infected insects, the oligoRING significantly decreased expression of the host *IAP-1* gene (*p* < 0.001) (Figure 5b). We also found significant down-regulation (by 36.6%) of baculovirus *IAP-3* and host *IAP-Z* genes on the 6th day (Figure 5a). It is almost impossible to measure expression of *vIAP-3* and *hIAP-Z* genes separately, since they are very similar, but their joint down-regulation directs cellular processes towards apoptosis. Surprisingly enough, their total expression was not significantly down-regulated on the 14th day. Of note, on the 14th day we did not find LdMNPV present in the surviving larvae in any of the experimental groups, which means that the death of all infected larvae occurred with complete eradication of LdMNPV in the insect organism is possible. The host *IAP-Z* gene increased its expression in the surviving larvae that have overcome viral infection (3.2 ± 0.8 times higher than mean value for control larvae).

### 2.4. Evidence for the Development of Apoptosis in oligoRING-Treated Insect Cells

**Figure 6:**
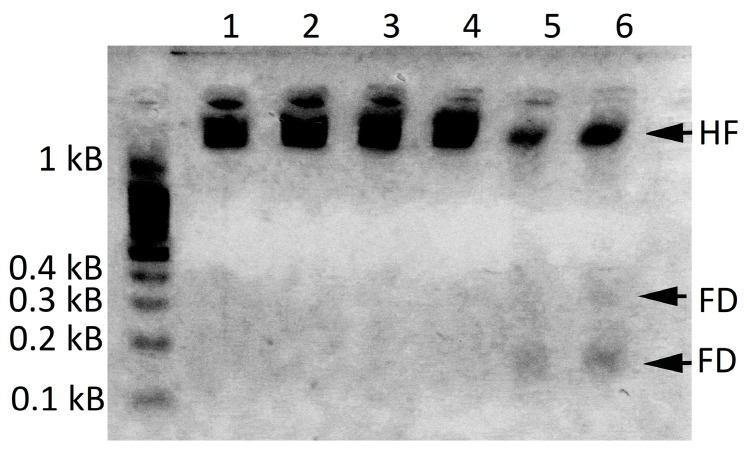
Electrophoretic separation of DNA extracted from tissues of live LdMNPV-infected gypsy moth larvae (1% agarose gels). The positions of DNA markers are indicated on the left. Lanes: 1,2—water control; 3,4—oligoCpG treatment; 5,6—oligoRING treatment. Signs: FD—apoptotic fragmented DNA (around 180 and 360 bp); HF—higher fraction of undergraded genomic DNA. Data in each agarose lane represent DNA from three live larvae from every group in the experiment.

We employed an additional approach, detection of apoptotic DNA ladders, to confirm that the oligoRING triggered higher levels of apoptotic processes in LdMNPV-infected cells, which subsequently led to a significantly higher mortality rate [29]. This well-established marker of apoptosis is the specific genomic DNA fragmentation of segments comprising a nucleosomal turn (~180 bp) [27]. Extraction of DNA was carried out with tissue from live insects on the 6th day of the experiment (which corresponds to the mortality peak in LdMNPV-infected larvae). Due to viral infection in dead LdMNPV-infected gypsy moth larvae, apoptosis as an antiviral response should be detected [30], which was confirmed in our preliminary study [29]. Here, we show that in live LdMNPV-infected larvae, only oligoRING-treated insects had two specific apoptotic DNA fragments (FD) of around 180 and 360 bp in length, respectively (Figure 6). This indicates the faster initiation of apoptotic degradation of genomic DNA in oligoRING-treated larvae compared with oligoCpG-treated larvae and controls.

In summary, the topical application of short antisense DNA insecticide from the LdMNPV *IAP-3* gene increases the mortality rate of LdMNPV-infected gypsy moth larvae and increases the insecticidal efficiency of the baculovirus treatment.

## 3. Discussion

This study reports progress made both in basic knowledge and in applied pesticide research. The results obtained from the DNA sequencing lead us to believe that we have found a fragment of the mRNA belonging to an anti-apoptosis gene (*IAP-Z* gene) of the gypsy moth. The oligoRING performed as a perfect complement to the base pairing on the fragment of gypsy moth cDNA and is an antisense molecule to the investigated mRNA. In this work, we provide evidence that the target gene, the *IAP-Z* gene, is definitely expressed and that oligoRING fragment down-regulates it in LdMNPV-free larvae. Its actions as an antisenseRNase H-dependent oligonucleotide [16] are accompanied by apoptotic patterns such as involution of cells, condensation (pyknosis), and fragmentation (karyorrhexis) of nuclear material. However, the observed effects may not have reached certain critical levels; the 2.04 ± 0.29-fold stronger down-regulation of host *IAP-Z* gene we observed may not be enough to cause significant mortality among the treated insects. In our previous experiments, some groups of LdMNPV-free gypsy moth larvae reared under laboratory conditions also did not show significant sensitivity to treatment with oligoIAPs [9]. This suggests that either insect populations may either possess different genetically based sensitivity to the specific oligonucleotide, or that an additional specific factor (or factors) might be necessary for apoptosis induction and synthesis of a sufficient amount of mRNA for a target anti-apoptosis gene silencing [3]. According to our theory, when the oligoRING shows a significant insecticidal effect on LdMNPV-free gypsy moth larvae, it may interfere with the expression of the host *IAP-Z* gene, which leads to apoptosis of the insect cells and subsequent death of larvae. Similarly, to observations of the RNAi in lepidopterans [3], the obvious reason why the oligoRING does not always exert an insecticidal effect on healthy and LdMNPV-free gypsy moth larvae grown under lab conditions compared with those grown in a natural habitat, with exposure to concomitant stress factors [8,9], is that its action depends on the dynamics of synthesis and breakdown of the mRNA of the target host *IAP-Z* gene. Although down-regulation of host IAP genes does not always lead to a significant mortality rate among LdMNPV-free larvae grown under laboratory conditions, in our experiments this approach always worked on early instar larvae insects of a younger larval age collected from a forest where the presence of many natural stress factors (baculovirus and bacterial infections, UV radiation, air pollution, etc.) may activate an apoptosis–anti-apoptosis system. For example, in this study we show that in LdMNPV-infected larvae 14 days post-infection, the total expression of host *IAP-Z* and LdMNPV *IAP-3* genes is 3.65 ± 0.39-fold more strongly up-regulated than the host *IAP-Z* gene in LdMNPV-free larvae (*p* < 0.05). This indicates the presence of a higher concentration of target mRNA molecules and increases the probability of interaction with the antisense oligoRING, leading to the down-regulation of expression of target anti-apoptosis proteins. Since gypsy moth control will mainly take place in forests, it is hypothesized that this approach will be of higher efficiency under natural conditions.

In the absence of stress factors (e.g., viral infection), concentration of the target anti-apoptosis mRNA would be low, and a pronounced insecticidal effect should not be generated by the oligoRING. In the case of LdMNPV-infected larvae, we think that the oligoRING obviously efficiently blocks mainly LdMNPV *IAP-3* mRNA and triggers higher levels of apoptosis in the infected cells, leading to the subsequent death of the insect. The high efficiency of baculovirus infection is explained in part by the ability of the virus to suppress the host defense machinery connected with the apoptosis pathway, an ability that accounts for baculovirus inhibitor-of-apoptosis genes (vIAPs). Indeed, in this study, LdMNPV-infected gypsy moth larvae were treated with an antisense oligoRING fragment specific to the viral *IAP-3* and host *IAP-Z* genes, and the oligoRING fragment caused significant total down-regulation of host *IAP-Z* and baculovirus *IAP-3* genes, which resulted in increased mortality of the insects and development of apoptotic DNA fragmentation. Thus, baculovirus infection in gypsy moths did not cause difficulties by silencing the nucleic acids described in [25], when we used an antisense oligonucleotide complementary to the virus *IAP-3* gene to avoid possible physiological barriers. To our knowledge, this is the first case of a recorded insecticidal effect of a virus-specific antisense oligoDNAs on LdMNPV-infected gypsy moth larvae, which could be of potential significance in insect pest management. We describe the newly discovered phenomenon using the term the VOVA (Virus before Oligonucleotide—Vent to Apoptosis) effect.

It is important to note that most of the baculovirus IAPs belong to the *IAP-3* group [28]. To achieve efficient infection rates, baculoviruses induce a pro-apoptotic DNA Damage Response (DDR) [31,32]. Induction of the DDR by viral replication increases virus yields up to 100-fold [31]. The DDR induces depletion of host inhibitor-of-apoptosis genes (hIAP), and thus, promotes cell death [33]. To overcome the consequences of DDR activation, baculoviruses employ special anti-apoptotic proteins (vIAPs) [10,12]. These proteins either interfere directly with the cellular apoptotic proteins, or alter the activity of cellular genes, leading to an anti-apoptotic state [34]. It is noteworthy that, despite the evolutionary relatedness of the vIAPs and hIAPs, there is an important structural distinction between them. In contrast to the hIAPs, which possess a specific N-terminal domain and are negatively regulated by signal-induced N-terminal degrons upon virus infection, the vIAPs do not have an equivalent N-terminal domain [14,33]. This makes vIAPs more stable and active as apoptosis inhibitors [14]. For the *Autographa californica* multicapsid nuclear polyhedrosis virus, it was found that by 12 h post infection, the viral transcripts comprised 38% of total cellular mRNA [35]. Thus, in the presence of LdMNPV infection, it is better to rely on the alteration of expression of functionally important virus genes (for example, anti-apoptosis genes) that could have an insecticidal effect on gypsy moths. According to data discussed here, the oligoRING fragment is among the antisense oligonucleotides most appropriate for triggering higher mortality among LdMNPV-infected gypsy moth larvae. By expanding our study from non-infected *Lymantria dispar* larvae to include LdMNPV-infected *Lymantria dispar* larvae, we were able to investigate the variants also to be found in nature, thus expanding the proofs of DNA insecticide effectiveness.

## 4. Materials and Methods

### 4.1. Origin of L. dispar Larvae

Egg masses of the gypsy moth *Lymantria dispar* (Lepidoptera: Erebidae) were identified and collected in November 2014 from forested wilderness located near Opolznevoye on the Crimean peninsula (lat. 44.3971, long. 33.9290, alt. 27.4 m).

### 4.2. Insect Rearing

Gypsy moth larvae were grown in Petri dishes under standard conditions with wheat germ-based medium at a temperature of 25 °C used for treatments [9,36,37,38]. Laboratory scales (Axis BTU210; Axis, Gdańsk, Poland) with 1 mg discreteness were used to weigh larvae.

### 4.3. Sequences of the Applied oligoDNAs Fragments

We designed an antisense RING domain fragment according to the LdMNPV genome sequenced by Kuzio et al. [13] found in ICTVdb database (http://www.ictvonline.org). The sequence of the RING domain fragment of the LdMNPV *IAP-3* gene is as follows: 5′-CGACGTGGTGGCACGGCG-3′ (antisense strand; experimental group; oligoRING). The sequences of oligoDNAs used as a control were as follows: 5′-CGCGCGCGCGCGCGCGCG-3′ (oligoCpG). OligoDNAs were synthesized by Evrogen (Moscow, Russia) [8,36].

### 4.4. OligoDNA Treatment of Lymantria Dispar Larvae

Before the experiment, larvae were randomized into groups and weighed to achieve the same starting point for measuring biomass for each group; the difference in biomass among different groups was within 3%. On average, 20–25 1st instar larvae were used per each control and experimental group for the treatment with oligoDNAsfragments. Each experiment was performed three times. A water solution with an ssoligoDNAsfragment (10 pmol/µL, either oligoRING or control oligoCpG sequence) was applied to larvae topically using a hand-held sprayer. We collected small drops of solution from the surface of 10 larvae and found approximately 0.2–0.3 µL solution on each larvae after pulverization (2–3 pmol of ssoligoDNAs per larvae). On the 7th and 14th day after the treatment, the biomass of live larvae was measured. To exclude possible LdMNPV infection, prior to experiments with non-infected gypsy moth larvae, we used PCR with two oligonucleotide primers specific to the LdMNPV capsid gene p39: 5′-ACGTTCTCGTTGAACGTGCTG-3′ (forward primer) and 5′-CTGGTGAACCACAAAACCCTG-3′ (reverse primer) [9].

### 4.5. Infection of Lymantria Dispar Larvae with LdMNPV

To infect the gypsy moth larvae with LdMNPV, the baculovirus preparation ‘Pinkvir’ (Pushkino, Russia) was used. After 1-day starvation, the insects were allowed to feed on the virus polyhedra-containing wheat germ-based medium for 2 days (10,000 virus polyhedra per 1 mg of medium). The larvae were then transferred to non-infected medium. Phase contrast microscopy and Goryayev’s chamber were used to count viral polyhedra in the ‘Pinkvir’ preparation. The larvae were treated with water, oligoRING, or control (oligoCpG) ssoligoDNAsfragment after 48 h infection with the virus.

### 4.6. Detection of the LdMNPV Infection in L. dispar by PCR

Specific PCR conditions and primers for the LdMNPV p39 capsid protein gene were used for detection of LdMNPV infection in gypsy moth larvae [9]. DNA was extracted using the DNA-sorb-AM kit (AmpliSens, Moscow, Russia) and PCR reactions were performed using the AmpliSens-200-1amplification kit (AmpliSens), following the manufacturer’s protocols. DNA was initially denatured for 3 min at 94 °C, followed by 5 cycles of 1 min denaturation at 94 °C, 1 min hybridization at 61 °C and 1 min elongation at 72 °C, followed by 30 cycles of 0.75 min denaturation at 94 °C, 0.75 min hybridization at 61 °C, and 0.75 min elongation at 72 °C, followed by a final elongation step at 72 °C for 5 min.

### 4.7. Search for L. dispar mRNA Homologous to the LdMNPV IAP-3 Gene

Total mRNA was extracted from non-infected gypsy moth larvae using an RNA Extract kit (Evrogen), following the manufacturer’s protocols. First strand cDNA synthesis was performed using a MMLV RT kit (Evrogen), following the manufacturer’s protocols. Primers oligoBIR (5′-GCCGGCGGAACTGGCCCA-3′, sense strand) and oligoRING (5′-CGACGTGGTGGCACGGCG-3′, antisense strand; used as a DNA insecticide in this experiment) from the LdMNPV *IAP-3* gene were applied to detect homologous anti-apoptosis mRNA in the gypsy moth using its first cDNA strand. PCR reactions were performed on 2 µL (10 ng/µL) of DNA using 0.75 units of Goldstar polymerase (Eurogentec, Angers, France), and 0.2 mM dNTP, 1.5 mM MgCl_2_, and 50 pmol of each primer. DNA was initially denatured for 4 min at 95 °C, followed by 30 cycles of 1 min of denaturation at 94 °C, 1 min of hybridization at 60 °C, and 1 min of elongation at 72 °C, followed by a final elongation step at 72 °C for 7 min. PCR products from the larvae were purified using the NucleoSpin Extract II Kit (Macherey-Nagel, Hoerdt, France) and the sequencing polymerase reaction was performed with Big Dye Terminator v3.1 RR-100 Mix (Applied Biosystems, Saint Aubin, France). Polymerase reactions were performed with 5 µL purified DNA and 0.35 µL of primers (10 pmol/µL). DNA was initially denatured for 2 min at 96 °C, followed by 30 cycles of 10 s of denaturation at 96 °C, 15 s of hybridization at 50 °C, and 4 min of elongation at 60 °C. Amplicons were sequenced in both directions with a capillary DNA sequencer (ABI PRISM 3100, Applied Biosystems). DNA sequences were analyzed using BLAST [39] and ClustalW 2.0.3 programs [40].

### 4.8. Quantification of Lymantria Dispar IAP-Z and IAP-1 Gene Expression

RNA extraction was carried out using a PureLink^®^ RNA Mini Kit (Ambion, Life Technologies, Waltham, MA, USA), according to the manufacturer’s instructions. For extraction, gypsy moth larvae were ground using a pestle in liquid nitrogen in 1.5 mL tube. Three independent extractions, each with two larvae, were carried out to produce replicates for each condition. The quality of the extracted total RNA was assessed by loading 5 µL of the eluted volume onto a 1.5% agarose gel and running the gel in TBE (Tris-borate-EDTA) buffer (10 V/cm) for 30 min. The quantity, intensity, and pattern of RNA bands were equal in all experimental groups, confirming the quality and reproducibility of RNA extraction from the insect material. For reverse transcription, the total RNA (5 µg) was annealed with oligo(dT)18 primer and analyzed using aRevertAid H Minus Reverse Transcriptase kit (Thermo Scientific, Waltham, MA, USA), according to the manufacturer’s instructions. The reaction was conducted at 42 °C for 60 min in Thermostat Termite (DNA Technology, Moscow, Russia).

For quantitative real time PCR studies and amplification with gene specific primers, an aliquot of obtained cDNA (0.5 µL) was used for each sample, with addition of the following primers for quantification of the *Lymantria dispar IAP-1* gene: forward 5′-CGCTGCAAGTAATGCTGAGG-3′, reverse 5′-GCACACGCAACTACATGTCC-3′ and *IAP-Z* gene, forward 5′-AGGCCCGTGTCGCCGGTC-3′ (oligo*IAP-Z*), reverse 5′-CGACGTGGTGGCACGGCG-3′ (oligoRING). The qPCRmix-HS SYBR (Evrogen) master mix was used according to the manufacturer’s instructions. A LightCycler^®^ 96 instrument (Roche, Basel, Switzerland) was used to set up amplification according to the following procedure: 10 min of initial denaturation at 95 °C, followed by 40 cycles with 10 s of denaturation at 95 °C, 20 s of annealing at 60 °C, and 16 s of elongation at 72 °C. As a final step, all PCR products were melted to estimate the specificity of amplification and presence of additional products.

### 4.9. Histological Studies

Histological slides were made using non-infected gypsy moth larvae 14 days after treatment with oligonucleotides. Live larvae were fixed in 10% formaldehyde for 2 days. After that, larvae were dried in isopropyl alcohol and then placed in paraffin blocks. Sections (4 µm) were made with a microtome (Rotmik, Moscow, Russia), stained with hematoxylin and eosin (BioVitrum, Sankt-Peterburg, Russia), and fixed for analysis according to the manufacturer’s instructions (Blik, Sankt-Peterburg, Russia).

### 4.10. Apoptosis Detection

A Quick Apoptotic DNA LadderDetectionKit (Life Technologies) was used according to the manufacturer’s instructions to investigate the level of apoptotic DNA fragmentation in live LdMNPV-infected gypsy moth larvae.

### 4.11. Statistical Analyses

We used the non-parametric Pearson’s chi-squared test (χ^2^) with Yates’s correction and the Mann–Whitney test to evaluate the significant difference between the groups’ means (STATISTICA 7 software, Palo Alto, CA, USA).

## 5. Conclusions

The results obtained suggest that in LdMNPV-free and LdMNPV-infected gypsy moth larvae, the oligoRING acts as an antisenseRNase H-dependent oligonucleotide inducing the degradation of target mRNA of the host *IAP-Z* and LdMNPV *IAP-3* genes, followed by subsequent down-regulation of target protein expression. Results obtained for insect mortality, biomass accumulation, histological studies, and analysis of DNA apoptotic fragmentation suggest that, while the oligoRING induced apoptotic processes in both LdMNPV-free and LdMNPV-infected insect cells, they were more pronounced in the latter.

Data show the possibility for the joint use of very short (18 nucleotides) antisense fragments of the baculovirus anti-apoptotic genes and LdMNPV preparations to control gypsy moth propagation and further spread, thus providing evidence for this principle in insect pest management.

## Figures and Tables

**Table 1:** Statistical analysis of the insecticidal effect of oligoDNAs on LdMNPV-free and LdMNPV-infected gypsy moth larvae. Pearson’s chi-squared test (χ^2^) with Yates’s correction was used to evaluate the significant difference in larval death for each treatment (three replicates per treatment). χ^2^ values were calculated for experimental groups versus water-treated control groups on the 14th day after the treatment. ** indicates significance when *p* < 0.01.

Experimental Groups		*χ^2^* Values	Total Number of Larvae in Two Compared Groups
LdMNPV-free	oligoCpG vs. water control	1.11	150
	oligoRING vs. water control	0.08	151
LdMNPV-infected	oligoCpG vs. water control	0.05	117
	oligoRING vs. water control	9.63 **	118

## Supplementary Materials

Supplementary materials can be found at https://www.mdpi.com/1422-0067/18/11/2446/s1.
