# Supplementary material for: Molecular Alliance of Lymantria dispar Multiple Nucleopolyhedrovirus and a Short Unmodified Antisense Oligonucleotide of Its Anti-Apoptotic IAP-3 Gene: A Novel Approach for Gypsy Moth Control
Source: Int J Mol Sci. 2017 Nov 17;18(11):2446. doi: 10.3390/ijms18112446 (PMC5713413; doi:10.3390/ijms18112446)
Supplement: Supplementary file 1 [file ijms-18-02446-s001.pdf]

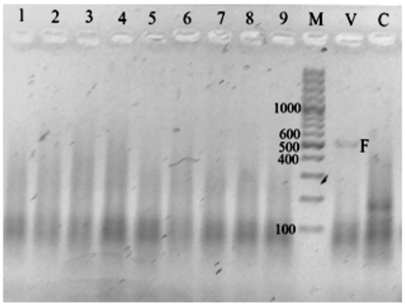

Supplemental Figure S1. Electrophoregram of DNA amplification products of gypsy moth: C – control; M – Standard DNA ladder (1 kb); 1-9 – DNA spectra of selected individuals + primers for detection of LdMNPV; V (positive control) – LdMNPV ('Pinkvir,' Russia) + primers for detection of LdMNPV; F – fragment of LdMNPV genome 524 bp long.

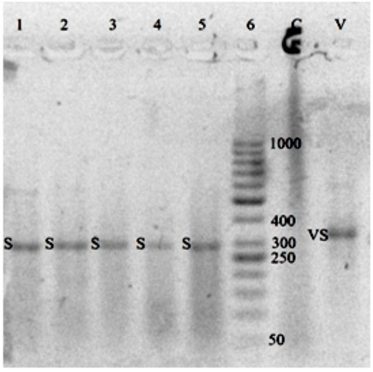

Supplemental Figure S2. Electrophoregram of DNA amplification products (with BIR and RING domain fragments of LdMNPV IAP-3 gene as primers) based on cDNA derived from gypsy moth mRNA. Lines 1, 2, 3, 4, 5 – electrophoretic DNA spectra of selected gypsy moth; line C – control; line 6 - standard 50 bp DNA ladder; line V – electrophoretic DNA spectrum of LdMNPV (the baculovirus preparation 'Pinkvir,' Russia). Sign S – target DNA fragment of gypsy moth larvae taken for sequencing (around 290 bp); sign VS – target DNA fragment of virus taken for DNA sequencing (length predicted according to genome sequence from ICTVdb is 317 bp).

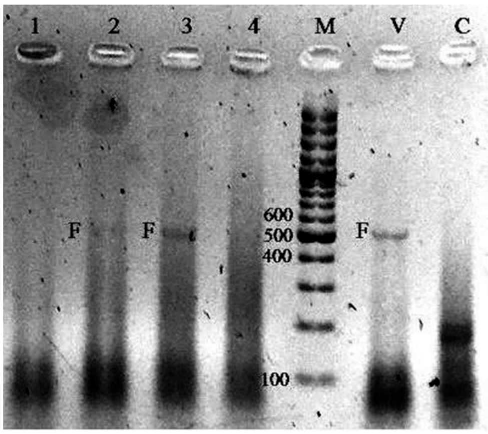

Supplemental Figure S3. Electrophoregram of DNA amplification products of representative LdMNPV-infected gypsy moth larvae: C – control; M – standard DNA ladder (1 kb); 1-4 – electrophoretic DNA spectra of LdMNPV-infected individuals + primers for detection of LdMNPV; V (positive control) – LdMNPV ('Pinkvir,' Russia) + primers for detection of LdMNPV; F – fragment of LdMNPV genome 524 bp long.
